# Effect of Cement Layer Thickness on the Immediate and Long-Term Bond Strength and Residual Stress between Lithium Disilicate Glass-Ceramic and Human Dentin

**DOI:** 10.3390/ma14185153

**Published:** 2021-09-08

**Authors:** João Paulo Mendes Tribst, Alison Flavio Campos dos Santos, Giuliane da Cruz Santos, Larissa Sandy da Silva Leite, Julio Chávez Lozada, Laís Regiane Silva-Concílio, Kusai Baroudi, Marina Amaral

**Affiliations:** 1 Department of Dentistry, University of Taubaté (UNITAU), Taubaté 12020-340, Brazil; flaviosantosdr@outlook.com (A.F.C.d.S.); giucs90@gmail.com (G.d.C.S.); larissasandy.ls@gmail.com (L.S.d.S.L.); regiane1@yahoo.com (L.R.S.-C.); d_kusai@yahoo.co.uk (K.B.); marinamaral_85@yahoo.com.br (M.A.); 2 Department of Operative Dentistry, FO-National University of Córdoba, Córdoba 5016, Argentina; juliochavezlozada@gmail.com

**Keywords:** dental bonding, polymerization, finite element analysis, dental materials

## Abstract

This study tested whether three different cement layer thicknesses (60, 120 and 180 μm) would provide the same bonding capacity between adhesively luted lithium disilicate and human dentin. Ceramic blocks were cut to 20 blocks with a low-speed diamond saw under cooling water and were then cemented to human flat dentin with an adhesive protocol. The assembly was sectioned into 1 mm^2^ cross-section beams composed of ceramic/cement/dentin. Cement layer thickness was measured, and three groups were formed. Half of the samples were immediately tested to evaluate the short-term bond strength and the other half were submitted to an aging simulation. The microtensile test was performed in a universal testing machine, and the bond strength (MPa) was calculated. The fractured specimens were examined under stereomicroscopy. Applying the finite element method, the residual stress of polymerization shrinkage according to cement layer thickness was also calculated using first principal stress as analysis criteria. Kruskal–Wallis tests showed that the ‘‘cement layer thickness’’ factor significantly influenced the bond strength results for the aged samples (*p* = 0.028); however, no statistically significant difference was found between the immediately tested groups (*p* = 0.569). The higher the cement layer thickness, the higher the residual stress generated at the adhesive interface due to cement polymerization shrinkage. In conclusion, the cement layer thickness does not affect the immediate bond strength in lithium disilicate restorations; however, thinner cement layers are most stable in the short term, showing constant bond strength and lower residual stress.

## 1. Introduction

When performing a ceramic restoration, the most recommended protocol is the use of resin-based cements combined to adhesive protocols during the cementation procedure [1,2]. This recommendation aims to achieve a clinical long-lasting bond between ceramic/resin cement and between resin cement/dental tissues [3,4]. In addition, the resin cements are easily handled, have an adequate setting time, and have the potential for both mechanical and chemical bonding [2,5].

However, the vertical misfit, or cement thickness, between the restoration and tooth preparation is an important factor which affects the success and survival of ceramic restorations [6]. The literature recommends a cement layer thickness around 50–100 μm for resin cements in ceramic crowns [7]. Furthermore, the bonding properties have been shown to be significantly reduced for cement thickness of 450–500 μm due to the residual stress of polymerization shrinkage [7,8]. May et al. [7] demonstrated a significant effect of the cement thickness on the failure loads of feldspathic ceramic crowns, showing that the cement layer thickness can be directly associated with the gap formation, increasing the tensile stresses on the crown’s intaglio surface and decreasing failure loads.

For that reason, several clinical reports have aimed to control the luting procedure and reduce the thickness of the cement layer by applying some kind of pressure during the restoration placement [9,10,11,12]. However, sometimes the beneficial effect of a thinner cement layer is not evidenced in these reports [9,10,11] and not always associated as an important factor in the clinical failures involving indirect dental restorations [13,14].

According to the literature, the cement space of ceramic crowns may vary for computer-aided design/computer-aided manufacturing (CAD/CAM) materials; additionally, there is no consensus on the best treatment option to improve the mechanical performance and bond durability. Previous clinical studies showed the mean internal adaptation of milled ceramic crowns ranged from 220 to 295 μm [15]. The mean discrepancies ranged from 137 to 175 μm for the same crown in different regions and from 148 to 203 μm for fixed dental prostheses [16,17]. A previous in vitro study evaluated the influence of occlusal resin cement space (50, 100, and 300 μm) on the fatigue performance of anatomical ceramic crowns bonded to a dentin analogue preparation [18]. According to the authors, the variation in the cement space did not affect the fatigue performance of CAD/CAM crowns [18]. Therefore, it is noticeable that previous studies have demonstrated the inverse relationship between the thickness of cement layer and bond strength, but this is not a consensus due to the wide variety of cement thicknesses considered in these previous reports. In addition, the evaluation of variation of cement thicknesses as an arithmetical progression could be useful to demonstrate how the linear increase in the thickness of cement layer could affect the bond strength values.

However, in addition to the residual stress, the exposed cement layer could expand by water sorption during the aging process [19] and therefore can present failures such as slow crack growth [20,21], which reduces the survival of composites and ceramics [14,15,16]. This phenomenon is responsible for the failure of the majority of dental biomaterials that are placed in the oral environment. Water sorption is also responsible for degradation of resin-based cements [22], and a thick marginal cement layer would be more exposed to the oral environment. In this sense, aging simulations in in vitro studies should be performed to elucidate the long-term bond strength achieved by dental materials and dental tissues [20,21,22,23]. Finite element analysis (FEA) is a numerical method that can be applied to elucidate the effect of polymerization shrinkage on stress; however, it was not performed in association with in vitro measurements of immediate and short-term bond strength. The association of this information could be useful to assist the comprehension of adhesive interface stability in restorative dentistry.

The aim of the present study was to evaluate the effect of different cement layer thicknesses on immediate and aged microtensile bond strength between lithium disilicate and dentin and to evaluate the residual stress of polymerization shrinkage according to the cement layer thickness using first principal stress analysis. The null hypothesis was that the cement layer thickness would not affect bond strength or residual stresses in the ceramic-dentin interface.

## 2. Materials and Methods

### 2.1. Sample Preparation

After approval of the university institutional ethical review board (Process n° 4.075.061), 24 first human molars donated from the university’s human teeth bank were embedded by root portions into chemically cured acrylic resin (JET, Classico, Cotia, Brazil) and had their occlusal surface flattened under constant cooling water using sandpaper #600 until dentin exposure was achieved. In sequence, the teeth were cleaned in ultrasonic bath with water for 10 min and stored until the luting procedure.

Lithium disilicate glass-ceramic blocks (IPS e.max CAD, IvoclarVivadent, Schaan, Liechtenstein) were sectioned with a low-speed diamond saw under constant cooling water (Isomet 1000, Buehler, Lake Bluff, IL, USA) to 24 blocks (6 × 6 × 7 mm^3^). The ceramic surfaces were ground flat with grit SIC papers (600, 800, and 1200 grit) using a polishing machine (EcoMet/AutoMet 250, Buehler, Lake Bluff, IL, USA) under cooling water. Then, the ceramic blocks were crystallized following the manufacturer’s instructions (850 °C/10 min). The blocks were randomly divided into three groups according to cementation weight (500 g, 1000 g or 3000 g) to obtain different cement layer thicknesses. For surface treatment, the ceramic blocks were etched with 10% hydrofluoric acid (Condacporcelana, FGM, Joinville, Brazil) for 20 s, rinsed with water, and dried with an oil-free air jet. Silane coupling agent (Monobond Plus, IvoclarVivadent, Schaan, Liechtenstein) was then applied on the surface with 60 s of volatilization time. 

The flattened dentin adhesive area was etched with 37% phosphoric acid for 15 s (Condac37, FGM, Joinville, Brazil), followed by a rinse of water for 20 s. The surface was dried with absorbent paper, and then the dental adhesive (Excite F DSC, IvoclarVivadent, Schaan, Liechtenstein) was applied and light cured for 15 s using the LED light curing device (BluePhase, IvoclarVivadent, Schaan, Liechtenstein). The luting procedure was performed with a dual cure resin cement (Variolink II, IvoclarVivadent, Schaan, Liechtenstein) following the manufacturer’s instructions. After positioning the ceramic blocks with resin cement on flat dentin, different loads (500, 1000 or 3000 g weight) were applied to the ceramic blocks to obtain different cement layer thicknesses. The excess cement was removed with a brush, and then light curing was performed for 40 s (BluePhase, IvoclarVivadent, Schaan, Liechtenstein), starting at the proximal margins on each side of the tooth.

After 24 h of storage into distilled water, 1 mm^2^ cross-section beams composed of ceramic/cement/dentin were obtained by means of a precision cutting machine (Isomet 1000, Buehler, Lake Bluff, IL, USA) under constant cooling water. The external beams of each block were delimited and removed.

### 2.2. Cement Thickness Measurement

Before testing the specimens, the cement layer thickness was examined by stereomicroscopy (Stereo Discovery V20, Zeiss, Gottingen, Germany), and three linear measurements in each sample were performed by a single calibrated operator. As standardization, each sample cement thickness average value was assumed as representative and considered a simplified homogeneous cement layer. Then, the samples (beams) were divided according to the cement layer in three different groups (*n* = 20) of thicknesses (60 μm [59.74 ± 8.41 μm], 120 μm [119.89 ± 21.85 μm] and 180 μm [182.66 ± 98.66]).

### 2.3. Microtensile Bond Strength (μTBS)

Half of the samples were considered baseline and were immediately tested, while the other half of the beams were subjected to storage in distilled water at 37 °C for 140 days for a posterior bond strength test. The final dimensions of each specimen were measured with a digital caliper and recorded.

To perform the μTBS, the specimens were glued to the testing device (OG01, Odeme, Lucerne, Brazil) with cyanoacrylate (Superbonder, Loctite, Dusseldorf, Germany). The setup was carried out in a universal testing machine (MBio, BioPDI, São Carlos, Brazil; 0.5 mm/min), and the bond strength (MPa) was calculated using the ratio between load at failure (N) and the adhesive area (mm^2^).

### 2.4. Assessment of Residual Polymerization Shrinkage Stress

**Figure 1:**
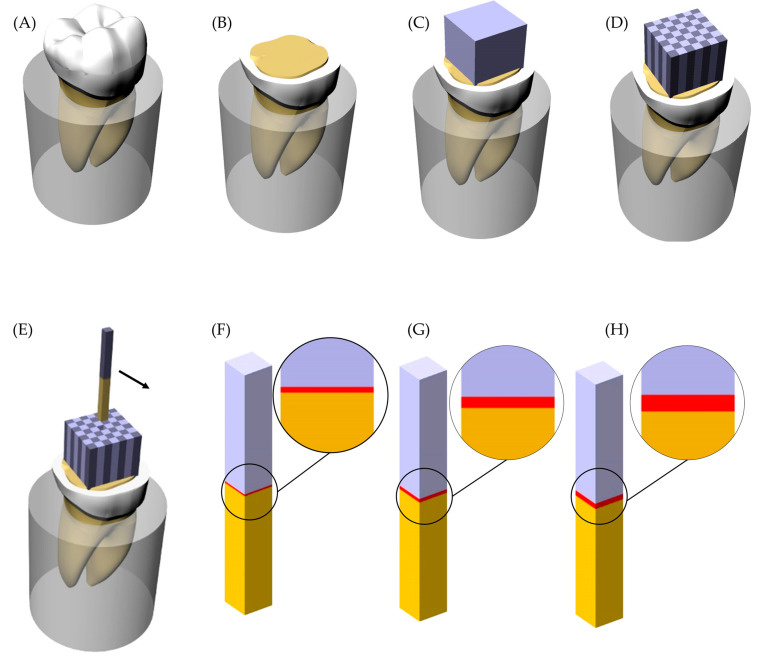
Sample preparation scheme and groups. (**A**) Sound tooth embedded into acrylic resin; (**B**) Flattened tooth with exposed dentin tissue; (**C**) Lithium disilicate glass-ceramic block cemented; (**D**) Sectioned sample with the beams separated; (**E**) Beam removed from the position; (**F**) Groups with 60 μm of cement layer thickness; (**G**) Groups with 120 μm of cement layer thickness and (**H**) Groups with 180 μm of cement layer thickness.

To assess the stress magnitude generated between the different cement layer thicknesses, the finite element method was applied. A three-dimensional (3D) model of an in vitro sample was modeled containing 8 mm of length with 1 mm^2^ of adhesive area. This model was replicated, and three different cement layer thicknesses were simulated in different models as well as the in vitro setup. The resultant Figure 1 summarizes the models considered in the present study. The geometries were imported into analysis software (ANSYS 19.2, ANSYS Inc., Houston, TX, USA) in STEP format (Standard for the Exchange of Product Model Data) and a mesh was generated using tet-10 element type. To reduce meshing error, a convergence test was performed to determine the appropriate mesh density (number of elements and nodes) with a threshold level set at 10% [24]. The material properties were assumed to be homogeneous, linear and with elastic behavior. The elastic modulus and Poisson ratio assigned for each material were derived from the literature (Table 1).

The external bases of the beam were fixed on the Z-axis (based in three-dimensional Cartesian coordinates oriented vertically). The adhesive interfaces were considered bonded. The polymerization shrinkage was simulated by thermal analogy, similar to previous reported FEA simulations involving polymeric dental materials [24,25]. The linear thermal expansion coefficient calculated was 0.005766. This information was inserted in the analysis software and temperature was reduced by 1 °C. A linear static structural analysis was performed to calculate stress magnitude in the dentin adhesive surface, cement layer, and lithium disilicate adhesive surface. The stress maps and peaks were recorded and tabled for the comparison between the models.

### 2.5. Data Analysis

**Figure 2:**
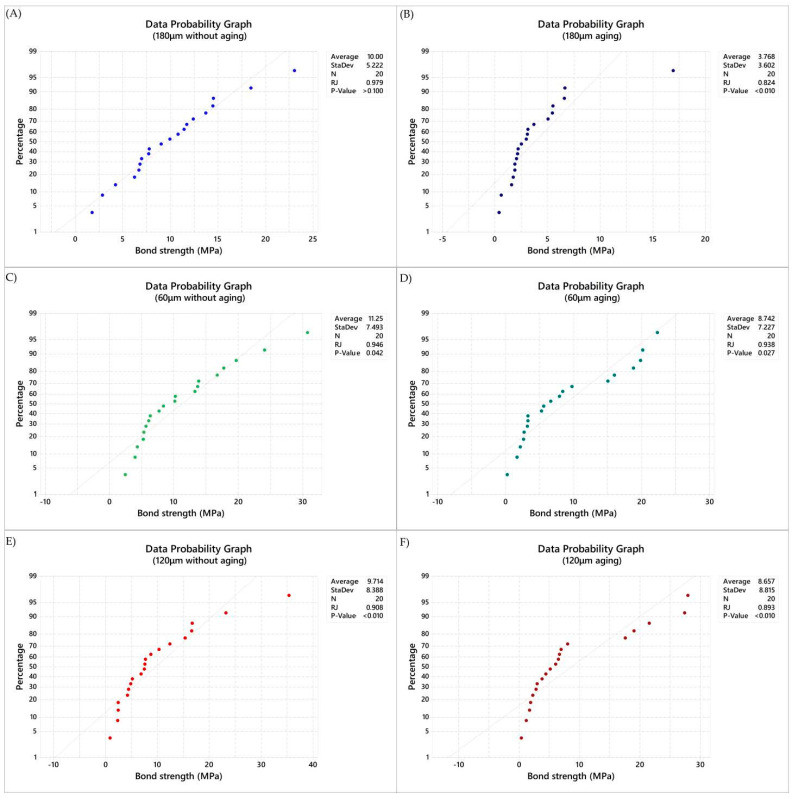
Normality data plot (Ryan–Joiner) applied in the present study. (**A**) Data probability graph in immediate tested samples with 180 μm of cement layer thickness, (**B**) Data probability graph in aged samples with 180 μm of cement layer thickness, (**C**) Data probability graph in immediate tested samples with 120 μm of cement layer thickness, (**D**) Data probability graph in aged samples with 120 μm of cement layer thickness, (**E**) Data probability graph in immediate tested samples with 60 μm of cement layer thickness, (**F**) Data probability graph in aged samples with 60 μm of cement layer thickness.

The bond strength (MPa) data were calculated, and the normality was rejected (Figure 2).

The μTBS results were statistically analyzed by Kruskal–Wallis and MINITAB Macro Dunn’s tests (α = 0.05) for both groups of samples: immediately tested and tested after aging simulation. This macro performs multiple comparisons in a nonparametric setting.

For that, the output was performed considering the number of comparisons (*k*),$\;k=\frac{k\left(k-1\right)}{2}$, the family alpha (α), the Bonferroni individual alpha (β),$\;B=\frac{\alpha }{k}$ and the 2-sided critical z-value.

The stress data (MPa) was qualitatively analyzed using the colorimetric stress maps and the stress peaks were used for the quantitative comparison assuming that values recorded from the same region with more than 10% of difference between the models are significant.

## 3. Results

The mean values of μTBS ranged between 11.24 and 3.76 MPa (Table 2). Kruskal–Wallis tests showed that there were no significant group differences (adjusted for ties) considering the immediate bond strength (*p* = 0.569).

However, the statistical test showed that the ‘‘cement layer thickness’’ factor significantly influenced the bond strength results for the aged samples (*p* = 0.028). Detailed statistical characteristic are summarized in Table 3. After post-hoc pairwise comparison, the aged groups showed significant differences between 60 and 180 µm cement thicknesses (*p* = 0.0125) and between 120 and 180 µm cement thicknesses (*p* = 0.0390).

**Figure 3:**
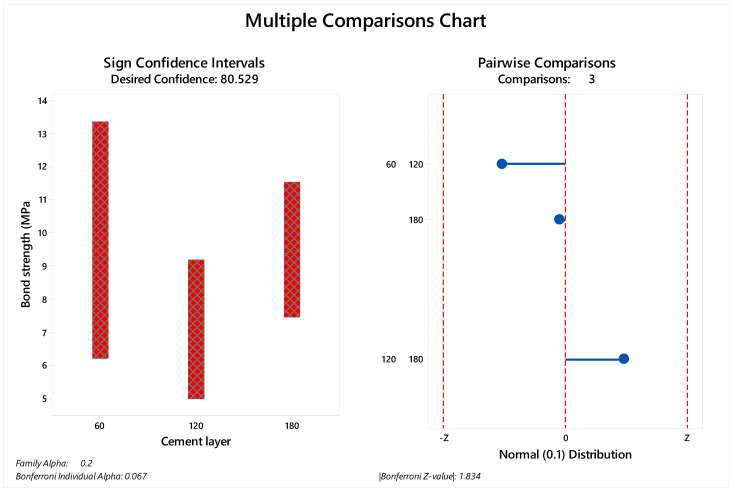
Multiple comparison chart for immediate tested groups (60, 120 and 180 µm). The sign of confidence was calculated considering family alpha = 0.2 and Bonferroni individual alpha = 0.067. The pairwise comparison demonstrated 1.83 as the Bonferroni Z-value without a visible difference between the evaluated cement layers.

**Figure 4:**
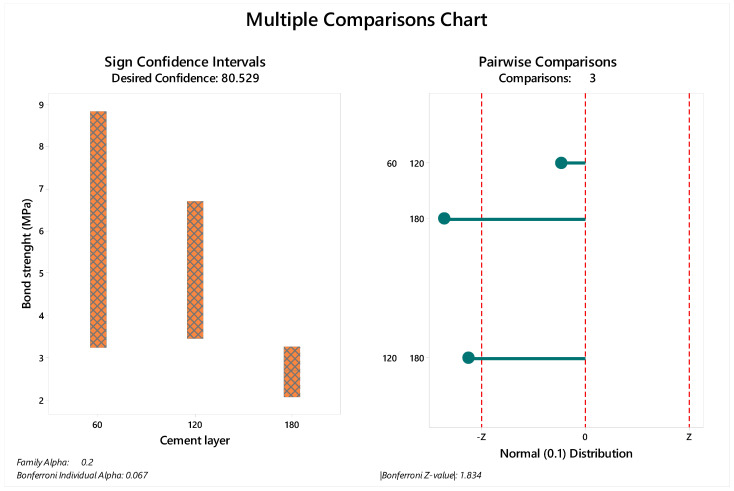
Multiple comparison chart for aged groups (60, 120 and 180 µm). The sign of confidence was calculated considering family alpha = 0.2 and Bonferroni individual alpha = 0.067. The pairwise comparison demonstrated 1.83 as the Bonferroni Z-value with visible differences between the evaluated cement layers.

The median and standard deviation of each value are summarized in Figure 3 for immediate tested groups and Figure 4 for aged groups. The bond strength data distribution considering sign confidence intervals and pair wise comparison can be observed in Figure 3 for the samples tested immediately after the cementation procedure and in Figure 4 for the aged samples. The achieved confidence calculated during the multiple comparisons statistic is summarized in Table 4 for immediate tested groups and Table 5 for aged groups.

**Figure 5:**
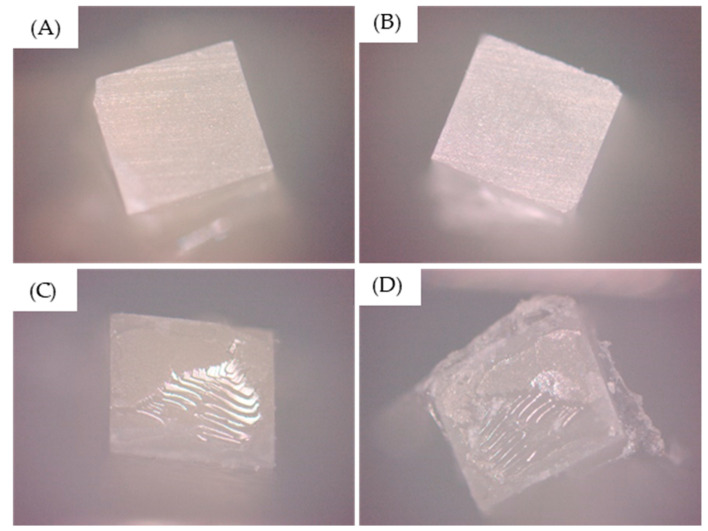
Predominant failure type observed after the testing. (**A**,**B**) Purely adhesive failures and (**C**,**D**) mixed failures.

Mixed failures (association of adhesive and cohesive failures) were predominant in all groups. The failure analysis is summarized in Figure 5.

**Figure 6:**
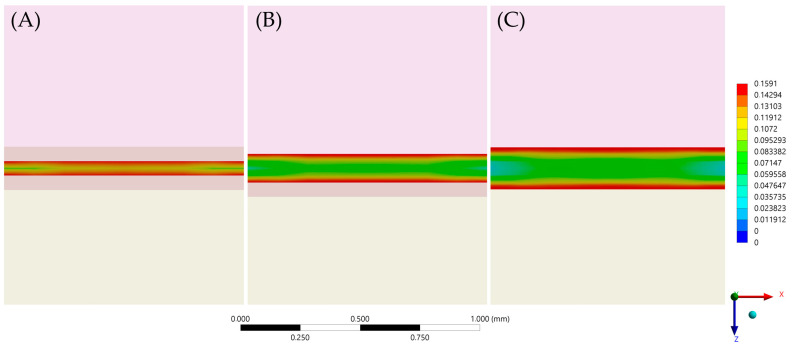
First principal stress (tensile) distribution in the cement layer for each evaluated group. (**A**) Models with 60 μm of cement layer thickness; (**B**) Models with 120 μm of cement layer thickness and (**C**) Models with 180 μm of cement layer thickness.

**Figure 7:**
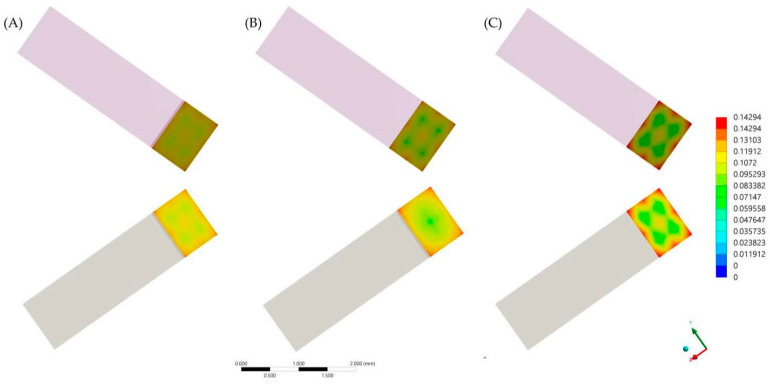
First principal stress (tensile) distribution in the ceramic (upper row) and dentin tissue (lower row) for each evaluated group. (**A**) Models with 60 μm of cement layer thickness; (**B**) Models with 120 μm of cement layer thickness and (**C**) Models with 180 μm of cement layer thickness.

After the numerical calculation process, the stress results (MPa) can be observed in the ceramic adhesive surface, cement layer and dentin adhesive layer which compose the adhesive interface (Figure 6 and Figure 7). In the cement layer (Figure 6), there is a visibly higher amount of stress with a higher volume of resin material concentrated in the bonded surfaces and with a lower magnitude at the center of the material. In the adhesive surface for dentin tissue and ceramic material (Figure 7), there is a similar stress pattern between surfaces from the same model; however, the higher the cement layer thickness, the higher the calculated stress magnitude (Table 6).

## 4. Discussion

The present study aimed to evaluate the effect of different cement layer thicknesses on immediate and aged microtensile bond strength between lithium disilicate and crown dentin, beside the residual stress of polymerization shrinkage. The results from both in vitro and in silico methods showed that the cement layer thickness can affect the bond strength and adhesive interface behavior. Therefore, the null hypothesis was rejected.

An adequate cementation procedure is a critical step to the success and longevity of ceramic restorations, since these biomaterials rely on adhesion not only for retention but also for resistance [12]. Unfortunately, the adhesive resin luting cement is a challenging procedure and involves multiple technique-sensitive steps [9,10,11,12]. Therefore, the present study demonstrates that the effect of a neglected cementation step will affect not the immediate but the long-term bond strength, probably compromising the restoration prognosis.

In addition to the bond strength, the fracture resistance of adhesively cemented ceramic restorations has been associated with the cement film thickness [26]. However, different parameters may affect the thickness of resin cement, and because of this, it is possible to observe reports showing average cement film thickness of 106.74 μm for the heat-pressing lithium disilicate ceramic and 340.35 μm for the milled CAD/CAM restorations. Despite the fact that the acceptable cement thickness of the International Standard Organization (ISO) is no more than 50 μm [26], there is a noticeable variation in the clinically achieved cement layer thickness.

In the present study, during the sample manufacturing process, the average cement thickness was in the range of 60 to 180 μm (Figure 1). It is possible to observe an indirect proportion between cement layer thickness and bond strength in literature [27]. In addition, a significant association between lower ceramic fracture load and thicker cement layer was already reported, indicating that a thin cement layer is more favorable for improved restoration mechanical response [27].

The stress maps calculated with the finite element method showed that the region of highest tensile stress magnitude was at the periphery of the cement layer; this formed in a centripetal behavior during the volumetric shrinkage. These results are in agreement with a previous study that found critical flaws at the margin of samples for μTBS [28] and also with a previous study that evaluated different resinous cement materials with a similar setup [28].

According to the literature, during the cementation procedure, the resinous cement should completely fill the space between the restoration and the tooth with no marginal discrepancy [29]. However, the cement film thickness can be strongly influenced by the type of luting cement and the seating force applied [29]. The present study applied different seating forces to obtain different values of cement thicknesses; however, it was necessary to verify each of the samples in the microscopy and to calculate the cement layer thickness with the analysis software before affirming the average cement layer per group.

Another reason that can justify the reduced bond strength in thicker cement layer group is the presence of porosity in the materials that could be relatively prominent in thicker layers of resin cement [27]. In addition, some authors proposed that a combination of surface preparation and the luting cement could act to move the fracture origin from the porcelain/cement interface to the cement surface [18,24,27].

The literature has reported that the lowest shrinkage stress (photoelastic analysis) was observed for the thinnest layer (25 µm) and proportionally increased with higher thicknesses (100 µm, 200 µm, and 400 µm) [30]. However, after the aging simulation, the authors reported that the use of thicker cement layers might have a positive clinical effect, resulting in the creation of expansion stress that could potentially influence the sealing of the marginal gap and enhance restoration-tooth retention [30]. This assumption was based on the expansion stresses found in the thinner cement layer group. The present study showed that there is a difference in the bond strength after the aging simulation regardless of the cement layer thickness, suggesting that the aging effect will be deleterious also for higher cement layer thicknesses and not only in the thinnest groups.

Thicker resin cement layers (verified by 3-dimensional microcomputed tomography) were also reported to promote higher polymerization shrinkage on stresses in ceramic [31]. At the same time, a thicker cement layer exposes more polymeric material to the oral environment, increasing its susceptibility to aging degradation. This suggested degradation susceptibility in larger areas of exposed cement could have contributed to the worst behavior found when 180 µm was considered, which in addition to the higher amount of residual stress, culminated in a significantly lower value of bond strength after aging in comparison with 120 µm and 60 µm layer thicknesses. The difference in stress peak in dentin tissue was approximately 13% between 120 to 180, while this same difference was approximately 1.5% between 120 µm to 60 µm. Therefore, it is possible to hypothesize that the stress state generated in the polymerization shrinkage is not a perfect linear regression as a function of the cement thickness.

The space planned for the resin cement layer in the digital workflow did not affect the fracture resistance of lithium disilicate veneers [32], simulating 120.4 µm, 174.9 µm (near the cement thickness simulated in the present study) and 337.2 μm of cement thicknesses. Therefore, the present study suggests that the use of thinner cement layers should be indicated to guarantee bond strength and adhesive interface integrity instead of improvements in the load-to-fracture values. The thickness of resin cement is considered a critical factor for the prognosis of indirect restorations. A greater thickness of cement increases the stress at the walls of the tooth cavity because of the polymerization shrinkage [33]. Therefore, not only could the bond strength be benefited with a thin cement layer, the cusp deflection could also be reduced in partial restorations. Thus, cement layer thickness has an important role in the mechanical behavior of adhesively cemented ceramic restorations. Further studies should be developed considering the bond strength with different ageing times, including lap shear, tensile and peel stresses evaluations.

## 5. Conclusions

During the restoration cementation procedure, a thicker cement layer thickness will not negatively affect the immediate bond strength. However, due to the higher volume of material, a higher magnitude of residual stress will be present and, during aging, the bond strength will be dampened. Therefore, to improve bond durability, thinner (60–120 µm) cement layer should be recommended.

## Figures and Tables

**Table 1:** Material properties considered to calculate the residual stress.

Material	Elastic Modulus (GPa)	Poisson Ratio	Volumetric Shrinkage (%)	Reference
Enamel	18	0.30	-	[25]
Lithium Disilicate glass-ceramic	95.0	0.30	-	[24]
Resin cement	7.0	0.24	1.74	[25]

**Table 2:** Means (in MPa) and standard deviations (±value) of the μTBS Test.

Cement Thickness (µm)	Immediate	After Aging
60	11.2 ± 7.4	8.7 ± 7.2
120	9.7 ± 8.3	8.6 ± 8.8
180	10.0 ± 5.2	3.7 ± 3.6

**Table 3:** Descriptive statistics from Kruskal–Wallis tests (MPa versus Cement layer).

Immediate				After Aging			
Cement (µm)	Median	Mean Rank	Z-Value	Cement (µm)	Median	Mean Rank	Z-Value
60	9.19	32.5	0.61	60	6.08	35.9	1.69
120	7.52	27.1	−1.06	120	5.54	33.5	0.94
180	9.45	31.9	0.45	180	2.69	22.1	−2.63
Overall		30.5		Overall		30.5	

**Table 4:** Confidence intervals, achieved confidence and data position according to the cement layer thickness for the immediate tested groups.

Cement Thickness (µm)	CI for η	Achieved Confidence	Position
60	(6.27; 13.21)	73.68%	(8; 13)
	(6.20; 13.37)	80.53%	Interpolation
	(6.04; 13.70)	88.47%	(7; 14)
120	(5.09; 8.65)	73.68%	(8; 13)
	(4.98; 9.18)	80.53%	Interpolation
	(4.75; 10.31)	88.47%	(7; 14)
180	(7.68; 11.43)	73.68%	(8; 13)
	(7.44; 11.52)	80.53%	Interpolation
	(6.92; 11.71)	88.47%	(7; 14)

**Table 5:** Confidence intervals, achieved confidence and data position according to the cement layer thickness for aged groups.

Cement Thickness (µm)	CI for η	Achieved Confidence	Position
60	(3.24; 8.42)	73.68%	(8; 13)
	(3.23; 8.84)	80.53%	Interpolation
	(3.23; 9.72)	88.47%	(7; 14)
120	(3.71; 6.64)	73.68%	(8; 13)
	(3.45; 6.71)	80.53%	Interpolation
	(2.90; 6.86)	88.47%	(7; 14)
180	(2.10; 3.10)	73.68%	(8; 13)
	(2.06; 3.27)	80.53%	Interpolation
	(1.99; 3.64)	88.47%	(7; 14)

**Table 6:** Stress peaks (in MPa) calculated * for each adhesive interface component.

Cement Thickness (µm)	Cement	Ceramic Adhesive Surface	Dentin Adhesive Surface
60	0.14	0.13	0.13
120	0.15	0.13	0.13
180	0.17	0.15	0.15

* Values obtained using the maximum probe software tool.

## Data Availability

Data available on request.
